# Targeting Drug Delivery System to Skeletal Muscles: A Comprehensive Review of Different Approaches

**DOI:** 10.1002/jcsm.13691

**Published:** 2025-02-05

**Authors:** Xiaofang Li, Jintao Xu, Shanshan Yao, Ning Zhang, Bao‐Ting Zhang, Zong‐Kang Zhang

**Affiliations:** ^1^ Faculty of Medicine School of Chinese Medicine The Chinese University of Hong Kong Hong Kong SAR China

**Keywords:** aptamer, internalisation, nanocarrier, peptide, skeletal muscle, targeting delivery

## Abstract

The skeletal muscle is one of the largest organs in the body and is responsible for the mechanical activity required for posture, movement and breathing. The effects of current pharmaceutical therapies for skeletal muscle diseases are far from satisfactory; approximately 24% of Duchenne muscular dystrophy (DMD) trials have been terminated because of unsatisfactory outcomes. The lack of a skeletal muscle‐targeting strategy is a major reason for these unsuccessful trials, contributing to low efficiency and severe side effects. The development of targeting strategies for skeletal muscle‐specific drug delivery has shown the potential for increasing drug concentrations in the skeletal muscle, minimising off‐target effects, and thereby improving the therapeutic effects of drugs. Over the past few decades, novel methods for specifically delivering cargo to skeletal muscles have been developed. In this review, we categorise targeting methods into four types: peptides, antibodies, small molecules and aptamers. Most research has focused on peptide and antibody ligands, and there are several well‐established drugs in this category; however, drawbacks such as protease degradation and immunogenicity limit their use. Aptamers and small molecules have low immunogenicity and are simple to chemically produce. However, small molecule ligands generally exhibit lower affinity because of their small size and high mobility. Aptamers are promising ligands for skeletal muscle‐targeting delivery systems. Additionally, if the active site of the cargo is located inside the cell, an internalisation pathway becomes necessary. The order of internalisation ligands and targeting ligands in the complex is a crucial factor, because an inappropriate order could lead to much lower targeting and internalisation efficiencies. Moreover, ligand density also merits consideration, as increasing the density of the targeting ligands may result in steric hindrance, which could impact the accessibility of the receptor and cause enlargement of the targeted ligands. More efforts are required to optimise drug delivery systems that specifically recognise skeletal muscle, with the aim of enhancing quality of life and promoting patient well‐being.

## 1 Introduction

Skeletal muscles, as one of the largest organs in the body, account for 30%–50% of the body weight and are responsible for the mechanical activity required for posture, movement and breathing [1]. Generally, skeletal muscle‐associated diseases can be classified into two main categories: primary muscular defects, such as muscular dystrophies (MDs), and disruptions of the regular homeostatic system that keeps the muscles healthy, such as metabolic and mitochondrial myopathies [2]. Skeletal muscle also participates in several metabolic diseases. For example, insulin resistance is one of the characteristics of diabetes and metabolic syndrome, and is related to lower insulin sensitivity in skeletal muscles [3]. It is therefore crucial to promote and maintain the health of skeletal muscles.

MDs are among the most prevalent genetic diseases in the world. Duchenne muscular dystrophy (DMD) is the most prevalent MD, which attracts most of the muscle‐related therapies. Patients with DMD lack functional dystrophin protein due to the deletions of dystrophin gene, leading to weakening muscle and death from respiratory or cardiac failure [4]. Recently, some therapies have been thoroughly studied and show promising outcomes in pre‐clinical and clinical trials for DMD [5, 6, 7, 8, 9], such as antisense oligonucleotide (ASO) and phosphorodiamidate morpholino oligomer (PMO) [10, 11]. ASOs and PMOs are synthetic, short, single‐stranded RNA molecules that attach to target RNA in a complementary manner, enabling them to affect RNA processing and modify protein expression [12]. In recent years, there are PMO drugs for specific exon skipping in patients with DMD, including Eteplirsen, Golodirsen, Viltolarsen and Casimersen, approved successively by the US Food and Drug Administration (FDA) [13, 14, 15, 16]. Long‐term investigations of these PMO drugs have indicated that these therapies reduce the deterioration of walking ability and maintain lung function in patients with DMD [13, 14, 15, 16, 17]. Although clinical trials have shown promising results, the effectiveness of PMO drugs in the target organ remains relatively inadequate [18]. Another primary obstacle in utilising PMOs is high renal clearance rate due to their neutrally charged nature [19]. Many vectors, such as adeno‐associated virus (AAV) and nanocarrier offer effective approaches for DNA plasmid and drug delivery in patients suffering from skeletal muscle diseases. Nevertheless, many non‐muscle tissues are also infected non‐specifically with AAV vectors [20]. The absence of specificity for muscle cells results in unanticipated detrimental effects throughout the body and a lower accumulation ratio. Thus, a skeletal muscle‐targeting delivery system is required to lower the dose, cost and side effects of current therapeutic drugs for skeletal muscle‐based diseases.

**FIGURE 1:**
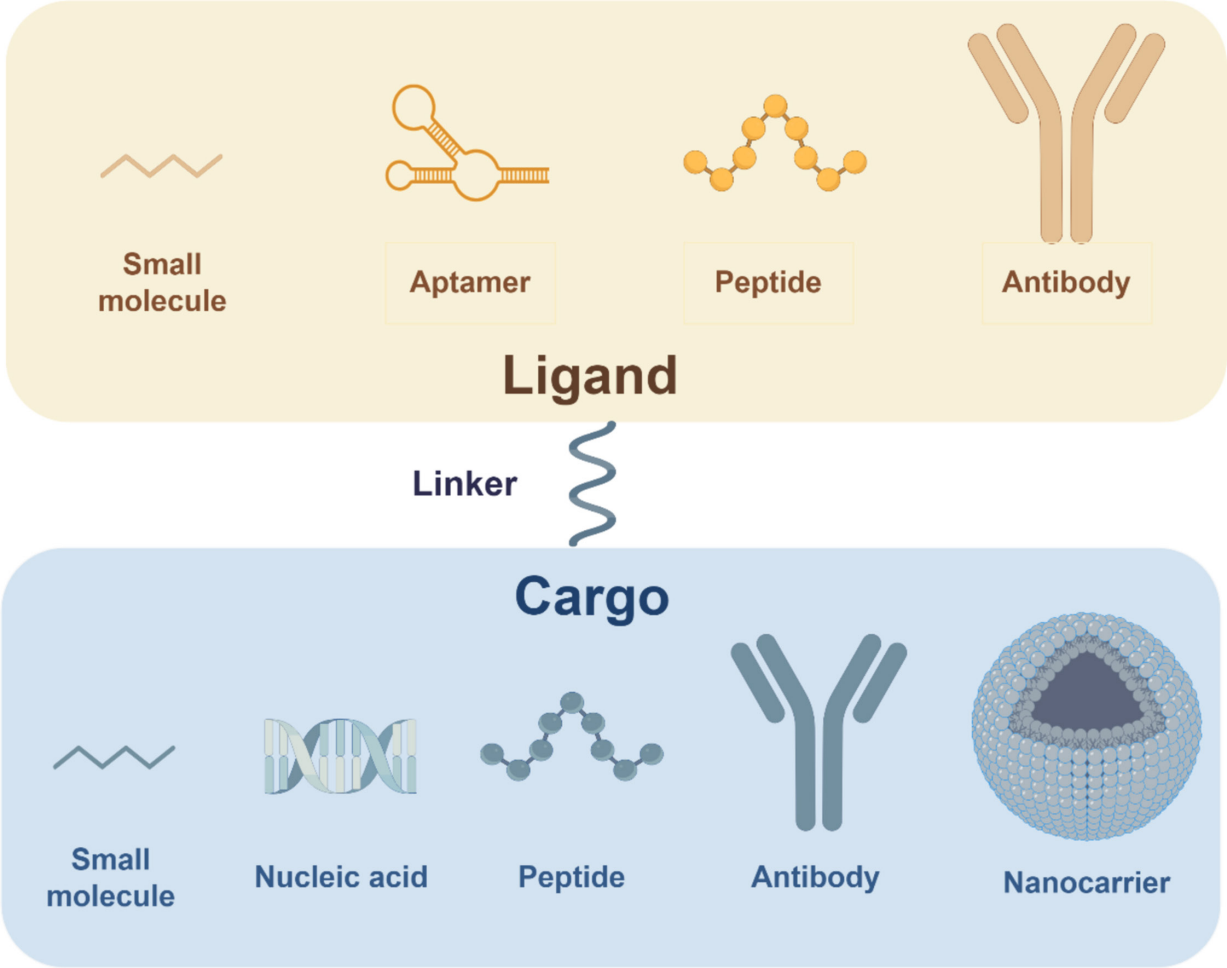
Schematic diagram of a targeted drug delivery system based on ligand, linker and cargo.

During drug development, safety concerns may arise due to off‐target activity in different tissues. One way to address this issue is to reduce the drug concentration in other tissues, especially those where safety concerns exist, and increase it in therapeutically relevant tissues. The use of tissue targeting ligands in the development of safe therapies has grown in popularity [2]. In this review, we focus on the skeletal muscle‐targeting delivery approaches and categorise the targeting methods into four types, peptides, antibodies, small molecules and aptamers (Figure 1). In the subsequent discussion, we provide an overview of the distinct characteristics, therapeutic advancements, and detailed screening methods for each strategy. The cell‐penetrating strategy is also the focus of our discussion, thereby providing generalised guiding design principles for skeletal muscle‐targeting drug delivery systems.

## 2 Muscle Targeting Strategies

Currently, different approaches are being used to achieve skeletal muscle‐targeting therapy. The first is targeted drug delivery, which recognises skeletal muscle‐specific cell surface elements and increases the drug concentrations in skeletal muscles relative to others. The other is based on specific intracellular components such as muscle‐specific promoters. Muscle‐specific promoters are specialised DNA sequences used in gene therapy to ensure that therapeutic genes are predominantly expressed in muscle tissues. Common examples include promoters derived from genes like skeletal muscle α‐actin, muscle creatine kinase, desmin and troponin I [21]. After the vectors containing muscle‐specific promoters and target gene sequences enter the cells, muscle‐specific promoters are recognised by transcription factors present in muscle cells. These transcription factors bind to the promoter region and initiate target gene transcription, leading to targeted gene therapy. Clinical trials based on various muscle‐specific promoters are ongoing [21]. The strategy of using muscle‐specific promoters aims to achieve gene expression in target cells, but the systemic distribution of drugs or vectors may result in adverse effects such as liver injury [22]. This review focuses on existing targeted therapies and novel skeletal muscle‐targeting delivery systems aiming for high distribution in skeletal muscle cells.

### 2.1 Antibody Targeting Strategy

Antibodies have been employed to specifically target various cells or tissues in the body, such as the brain [23], cancer cells [24, 25] or immune system cells [26]. Most antibodies have extended circulation periods lasting from several days to a few weeks. The long half‐lives of these antibodies result from their reutilisation by endothelial cells through surface receptors, which facilitates the exposure of a significant amount of therapeutic substances to the tissue [27].

One of the most commonly used cell‐surface receptors for antibody‐mediated drug delivery is the transferrin receptor 1 (TfR1). TfR1 is abundantly expressed in both skeletal and cardiac muscles, and antibody targeting this receptor delivers the coupled cargo to the cell by facilitating receptor‐mediated endocytosis [28]. AOC1001 (Avidity), a siRNA against myotonic dystrophy type 1 protein kinase (DMPK) RNA coupled to monoclonal antibody TfR1 (mAb TfR1), has been investigated for safety, tolerability, pharmacokinetics and pharmacodynamics at single and multiple‐doses in a Phase I/II clinical trial (NCT05027269) in 2023 [29, 30]. Another ASO drug coupled to a fragment of mAb TfR1, Dyne‐101, showed 40–50% splicing correction in the skeletal muscle and no harm after 13 weeks of treatment in cynomolgus monkeys. Now, Dyne Therapeutics is seeking volunteers for the clinical trial of Dyne‐101 (NCT05481879) [31]. In addition to mAb TfR1, mAb 3E10 also showed binding selectivity for skeletal muscle, and this specific binding may be related to myosin IIb in skeletal muscle [32]. Weisbart et al. constructed a micro‐dystrophin plasmid with the Fv fragment of mAb 3E10 and demonstrated its potential for use in the treatment of dystrophin‐deficient MDs [33].

Theoretically, the remarkable specificity of an antibody for its target antigen offers the possibility of highly selective skeletal muscle targeting. However, the existing mAb TfR1 showed muscle‐targeting properties, but in another study, mAb TfR1 was used to target the blood–brain barrier [34], suggesting that it does not possess high specificity for skeletal muscle tissue. Selecting an appropriate target antigen is one of the most crucial factors for improving the ability of antibody‐drug conjugation (ADC) to specifically target tissues. The target antigen must be abundant on the target cells, while having minimal or no expression in non‐target tissues, thus improving the precision of ADC‐targeting by restricting unintended interactions and buildup in non‐target areas [27]. Besides, antibodies, especially those derived from non‐human sources, can trigger immune responses in patients. Immunogenicity can lead to reduced drug efficacy and adverse effects. However, there are several strategies for reducing immunogenicity. First, replacing non‐human antibody regions with human sequences reduces immune responses [35]. Secondly, co‐administration of immunosuppressive drugs could help reduce the immune response against the antibodies. Furthermore, nanobodies, which are smaller fragments of antibodies, could be less immunogenic due to their smaller size and simpler structure [36]. Currently, the numbers of studies of muscle tissue‐targeting antibodies are fewer than those of peptides, and the reason for this may be related to their dimensions. Owing to the substantial dimensions of the antibodies, the loading efficiency remained low when a few small molecules were incorporated. Therefore, this method is restricted to specific drugs with high potency [27].

### 2.2 Peptide Targeting Strategy

Researchers have focused on the potential of small peptides to interact with particular cell types for drug administration and diagnostic imaging [37]. Several peptides have been designed to specifically target skeletal muscles. While the essential criteria for target receptors of peptide ligands are comparable to those of the target antigens of antibodies mentioned earlier, peptides as a targeting moiety exhibit several notable advantages: (i) they are smaller, allowing for better cell penetration; (ii) they can be easily synthesised using solid‐ or solution‐phase peptide synthesis methods and chemically modified; (iii) they are less likely to induce immune responses than viral vectors and antibodies; and (iv) they exhibit greater selectivity than conventional small molecules [2, 38].

Discovery tools, such as phage display and identification of important functional regions from known proteins, provide the opportunity to broaden the collection of peptide ligands that could bind to a certain target [27]. A phage is a protein‐coated virus made of DNA or RNA. A phage library is created by combining a random peptide/protein gene with a coat protein gene, which results in random peptide/protein expression on the phage surface. The process of biopanning is the affinity selection of phage libraries consisting of millions or billions of specially created phages against a target, which involves incubating the phage libraries with the target, washing away non‐binders, and isolating the binding phages [39]. In 1998, Samoylova et al. discovered a heptapeptide sequence, ASSLNIA, with improved skeletal muscle binding activity by screening a random phage display library using a combination of in vitro and in vivo selection [40]. The affinity of the ASSLNIA phage for skeletal muscle was increased by nearly five times compared with that of the control phage. At the same time, compared with the control phage there was a 75%, 65% and 42% reduction in ASSLNIA phage in the brain, liver and kidney, respectively. Additionally, a novel 12‐mer peptide (M12) was discovered through phage display screening in myoblasts to bind to skeletal muscle more preferentially than the liver [41]. In mdx mice (a mouse model of DMD, which carries a point mutation in the DMD gene resulting in a non‐functional dystrophin protein) administered PMO conjugated with M12, dystrophin expression was restored to approximately 25% of normal in the tibialis anterior, quadriceps, gastrocnemius, triceps and abdominal muscles, resulting in a significant improvement in grip strength. As reported by Gao et al., the ability of M12‐PMO to restore dystrophin expression was 10‐fold higher than that of an ASSLNIA‐PMO conjugate at an equal dose of 25 mg/kg and it showed no apparent harmful effects at the systemic administered dose of 75 mg/kg in mdx mice [41]. Besides phage display screening, targeting ligands can be selected based on the membrane constitution of skeletal muscle. Given that laminin binds to α‐dystroglycan (α‐DG), a protein present on the surface of muscle cells, Suzuki et al. conducted a screening process to identify peptides from laminin α2 chain LG4‐5 modules that bind to α‐DG. A specific peptide called A2G80 (VQLRNGFPYFSY) in the G domain of the laminin α2 chain, interacting with α‐DG, was observed among various synthetic laminin peptides [42]. Similarly, Furalyov et al. synthesised FS2 venom‐siRNA conjugation complex and the conjugation showed higher transfection effect compared with that of the control. The FS2 venom consists of 60 amino acids and specifically binds to L‐type Ca^2+^channels, which are muscle membrane proteins involved in the excitation and contraction of muscle fibres [43].

**FIGURE 2:**
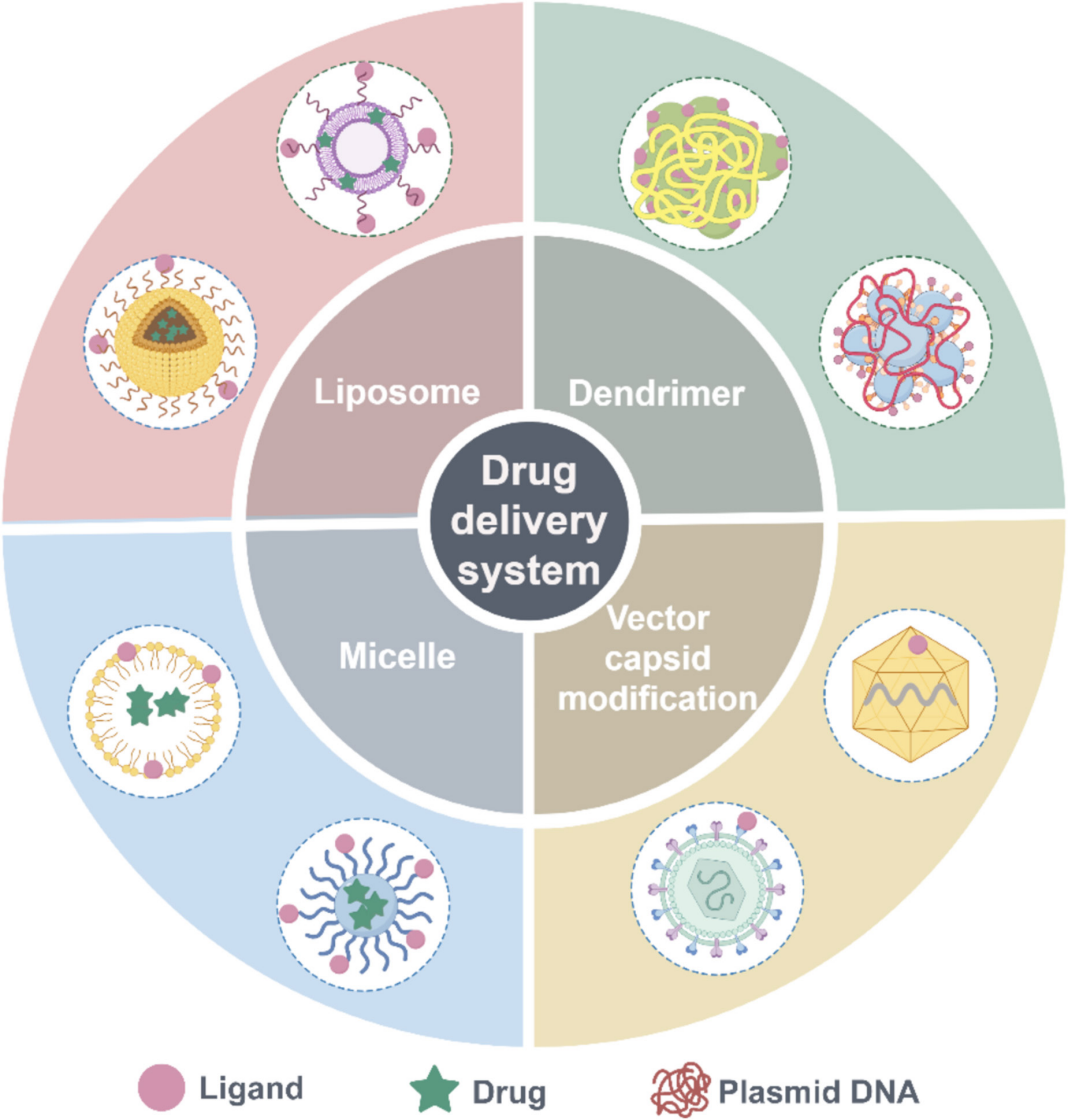
Schematic diagram of typical muscle‐targeting drug delivery systems based on nanocarriers and virus vectors.

Due to the simplicity of chemical modification of peptides, targeting peptides could be directly attached to medicines to create peptide‐drug conjugates (PDC), or conjugated with vectors to produce peptide‐modified nanocarriers, or linked to virus capsid genes resulting in peptide‐modified viral vectors (Figure 2) [44, 45]. Ideally, novel drug carriers offer improved drug solubility and stability, and mediate sustained or controlled drug release [46]. Muscle‐targeting peptides as drugs carriers could offer the tissue specificity and a dose reduction. For instance, when the previously mentioned peptides A2G80 or ASSLNIA were added to the surface of micelles or liposomes, the modified nanoparticles accumulated more effectively in the muscle tissue than in the control [47, 48]. Nanocarriers modified with targeting peptides may be a helpful tool for drug delivery. Muscle‐targeting peptides identified by phage display screening were primarily linked to the drug using linkers in a 1:1 ratio. When targeting peptides are conjugated with nanocarriers, this ratio may increase because of the high encapsulation efficiency. This innovative delivery method utilising nanoparticles and muscle‐targeting peptides offers a promising opportunity for the advancement of long‐term therapeutic approaches for the treatment of skeletal muscular diseases.

Despite the greater diversity of peptide libraries in phage display technology and the initial confirmation of the feasibility of AAV capsid modification with peptides [49], two potential issues may arise when incorporating ligands discovered using phage display technology into the AAV capsid. First, incorporating the ligand into the AAV capsid may render the protein non‐functional. Another issue is the ligand may not successfully translated into the heterologous structure of the AAV [50]. In order to overcome the issues of muscle targeting in a specific context, Ghosh and Barry created phage libraries that are “context‐specific” by incorporating the H and I β sheets of the Adenovirus 5 (Ad5) fibre onto the pIII protein of a filamentous bacteriophage [51]. A 12‐amino‐acid random peptide library was substituted for the HI loop between the sheets, which were held together by disulfide bonds. Peptide 12.51 screened from a context‐specific phage display library had a higher affinity for C2C12 myoblasts than the benchmark peptide. After translating peptide 12.51 back into the knob domain of Ad5, the peptide exhibited a 14‐fold increase in transduction on C2C12 myoblasts and a 2‐fold increase on differentiated C2C12 myotubes [51]. Later, Nguyen et al. showed that context‐specific phage display library had the potential to be applied to additional insertion sites apart from the HI loop, such as the hypervariable region (HVR) 5 loop of the Ad5 hexon protein [50]. Apart from context‐specific phage display, Weinmann et al. proposed a flexible methodology that allowed for the direct and simultaneous evaluation of specific AAV capsids in a large‐scale manner within the same animal [52]. This was achieved by integrating DNA/RNA barcoding with next‐generation sequencing (NGS), carrying out multiplexed secondary screening directly in animals and significantly decreasing the number of animals and the time of finding most potential AAV variants in clinical. Based on this, a modified form of AAV9, named AAVMYO, was identified, which presented peptides with exceptional selectivity in skeletal muscle when administered peripherally [52]. Moreover, Tabebordbar et al. created the DELIVER strategy, which involved using the expression of transgenic RNA to guide the evolution of AAV capsids in vivo. This approach allowed for the generation of a diverse library of capsids and their selection based on stringent transcript‐based criteria. It also enables the identification of functional capsid variants in any tissue of interest or animal model [53].

Despite the effectiveness of AAV vectors, they do have some notable shortcomings, including pre‐existing immunity to AAV capsids, limited packaging capacity, poor transduction efficacy in specific tissues, dose‐dependent toxicity and manufacturing challenges [54]. Much research is now devoted to these issues. For example, utilising different AAV serotypes that the patient has not been exposed to can help bypass pre‐existing immunity [55]. Besides, immunosuppressive drugs and plasmapheresis could help reduce the immune response [56, 57]. Overall, AAV is an important vector for gene therapy for skeletal muscle diseases, and the addition of targeting peptides could enhance the efficacy of AAV treatment.

In addition to peptides, some proteins can be used to modify viral or non‐viral vectors. Myomaker and Myomerger, (also known as Myomixer and Minion), are proteins responsible for regulating the fusion of precursor cells to create multinucleated myofibres during skeletal muscle growth and regeneration [58]. Hindi et al. achieved targeted delivery to skeletal muscle through the membrane of encapsulated viruses modified with Myomaker and Myomerger [58]. It was shown that the introduction of viral particles modified with muscle fusion proteins, either locally or systemically, could effectively transport micro‐dystrophin to the skeletal muscle of a mouse model of DMD, resulting in the alleviation of pathological symptoms. Similarly, Myomaker and Myomixer can be used to modify extracellular vehicles (EVs) coupled with the myotropic transmembrane proteins M‐cadherin [59].

Although muscle‐targeting peptides have a decreased tendency for immunogenicity compared with antibody‐targeting techniques, oral bioavailability is still expected to remain relatively low [2]. Similar to antibodies, peptide‐based targeting ligands also face additional hurdles due to protease degradation [27]. Moreover, the phage display method for peptide selection can be labourious, and it is well acknowledged that some false‐positive peptides (such as amplification bias or binding to plastic) may be identified and mistaken for targeting peptides. However, the likelihood of success is significantly enhanced by assessing the phage display results using NGS. NGS enables the use of a single screening cycle, thus limiting the dominance of parasite peptide sequences and facilitating the detection of sequences with greater ease and reliability [60]. Furthermore, it is important to consider the selection of targeting peptides that are most effective when used in combination with specific drugs. The choice and density of the targeting ligands may also affect drug potency through their spatial structures.

### 2.3 Small Molecule Targeting Strategy

Owing to the ease of their chemical conjugation to their medicinal payloads, small compounds are among the first targeted ligands to be investigated in many fields [27]. The average molecular weight of small molecule ligands is below 1 kDa [61]. Small molecules are often easier to manufacture, which increases their usefulness. Although many cancer‐targeting small‐molecule agents have completed clinical trials [27], the number of muscle‐targeting drug carriers based on small molecules under development is far lower. Previous studies have revealed that hydrophobic components could enhance the potency of antisense oligonucleotides in muscles [10, 11]. Benichou et al. demonstrated that in myotonic dystrophy type 1 (DM1) model mice, the distribution of C16‐HA‐ASO (a conjugation of palmitate hexylamine phosphodiester and IONIS‐486178 ASO) into the striated muscles was dramatically increased compared with that of ASO after systemic injection of 25 mg/kg. Additionally, C16‐HA‐ASO was more effective than ASO at lowering mutant hDMPK transcripts in the skeletal muscles of the animal model by up to 92%. Muscle strength increased significantly in correlation with the C16‐HA‐ASO‐induced reduction in mutant hDMPK transcripts in skeletal muscles. Meanwhile, C16‐HA modification reduced the distribution of ASO to the kidneys [62]. Similar to peptide‐modified nanocarriers, small molecules could also be conjugated with nanoparticles. Based on the high affinity of carnitine for the Na+‐coupled carnitine transporter (OCTN), which is highly expressed on skeletal muscle cell membranes, nanoparticles were conjugated with an amphiphilic derivative of L‐carnitine to actively target skeletal muscle cells. Compared with untargeted‐nanoparticles, the conjugated nanoparticle showed a higher concentration in myotubes [63].

Currently, the number of small molecules used for skeletal muscle targeting is far fewer than the numbers of peptides and antibodies. Most small‐molecule screening techniques rely on structure‐based rational drug design and assisted virtual screening methods, which have a slower progression than peptides and antibodies. The main obstacles faced by small‐molecule targeting ligands are their high mobility, which can lead to off‐target effects. Additionally, due to their size, small molecule ligands generally exhibit lower affinity, although exceptions to this rule may exist. However, from another perspective, the small dimensions, simplicity of chemical production and low immunogenicity render small compounds highly advantageous for targeting purposes [27].

### 2.4 Aptamer Targeting Strategy

Another representative low‐molecular‐weight targeting ligand is the aptamer. Aptamers are artificial, short (15–100 nt), single‐stranded nucleic acids (ssDNA or RNA) and have unique secondary structures. Aptamers possess numerous benefits, such as a wide array of targets (ranging from cells to metal ions), straightforward manufacturing and modification, minimal toxicity and immunogenicity, compact size and excellent durability under multiple environmental conditions [64]. As targeting ligands for drug delivery, a number of versatile functional groups can be chemically or enzymatically added to aptamers to enable bioconjugation with drugs or to optimise the biostability of the aptamer. An example of this is an aptamer chemically modified with 20‐fluoropyrimidines (20‐F) to decrease its nuclease sensitivity [64]. Aptamer drug conjugations (ApDCs) have been investigated for several therapeutic approaches, including chemotherapy, immunotherapy, radiation and phototherapy, and are utilised in several diseases based on disease‐specific biomarkers, including cancer and acquired immune deficiency syndrome (AIDS) [65].

Aptamers can be discovered using the Systematic Evolution of Ligands by Exponential Enrichment (SELEX) technique [66]. Through a series of affinity purification and amplification rounds, the most promising candidate aptamer sequence can be selected from a large pool of random sequences [64]. SELEX technology has been expanded with variations such as cell‐SELEX, crossover SELEX and tissue‐SELEX. These approaches enable the binding of a wide range of targets, including chemical molecules, nucleotides, proteins and whole cells and organisms [64]. For example, Philippou et al. discovered a skeletal muscle‐specific RNA aptamer A01B after 15 selection rounds using a cell‐internalisation SELEX method. The A01B RNA aptamer selectively entered skeletal muscle cells and showed a reduced binding affinity for non‐target cells. In contrast, the scramble control predominantly remained localised in the area between the myofibres [64]. Additionally, A01B could be conjugated with nanoparticles to enhance the drug shelf life, increase the medication loading capacity and optimise passive drug delivery efficiency [67, 68]. HSM01 is a human skeletal muscle cell‐specific ssDNA aptamer that showed a Kd of 109.5 nM in the binding dynamic curve. Under confocal microscopy, HSM01 was found to be located in the cell membrane and cytoplasm of human skeletal muscle cell, whereas the control was poorly attached and invisible [66]. HSM01‐linked nanoliposomes were created to improve the performance of aptamers in targeted drug delivery. The diameter of Lipo‐PEG‐apt was approximately 100 nm and it showed good homogeneity. The liposome encapsulation rate (LE) and liposome drug‐carrying rate (LC) were both 62.5%. When Lipo‐PEG‐apt complexes were injected into tree shrews via the tail vein, the aptamer‐linked liposomes primarily targeted the skeletal muscle, particularly in the limbs and abdominal muscle tissues. Furthermore, at the same concentration, Lipo‐PEG‐apt exhibited no significant side effects on the histological structure or inflammatory indicators in the liver and kidneys of rats [66].

In general, aptamers are multifunctional, flexible, three‐dimensional structures that can be coupled to a wide range of therapeutic agents and nanocarriers to enable their cell and deep‐tissue delivery [27]. Aptamers are produced using a controlled laboratory procedure, exhibit minimal variation across batches, and, unlike antibodies, do not require biological systems for production [67], minimising the risk of bacterial or viral contamination. Additionally, the relatively low molecular weights of aptamers and ApDCs make them a promising option for achieving faster and deeper tissue penetration compared with ADCs [65]. Aptamer‐targeting systems do, however, come with certain inherent difficulties. The biggest obstacle is their degradation in biological media because they are essentially nucleic acids. Aptamers may be eliminated quickly via renal filtration before they meet their targets. One strategy to overcome this problem is to modify nucleotide bases. The integration of nanotechnology into aptamers is another method that could help address this problem, enhance drug‐loading capacity and optimise passive drug delivery efficiency. Finally, an aptamer may still exhibit some cross‐reactivity despite its specificity. To address this difficulty, strict selection criteria and synthesis techniques should be used in the future [27].

## 3 Internalisation Strategies

Although the majority of studies have shown that the addition of targeting ligands results in enhanced accumulation and effects of the cargo in target tissues, a few results have indicated that the exon skipping efficiency of a complex of drug and muscle‐targeting ligand is not better than that of the drug alone, possibly due to lack of cell internalisation [69]. After the complex is released into the bloodstream, it needs to reach the target tissue and attach to the therapeutic target. Some targeting ligands can only “guide” the complex to the surface of the target cells and tissues. For a drug to exert the desired effect, it must be internalised if the therapeutic target is to be located within the cell. The insufficient ability of a complex to passively cross a barrier requires an active uptake process [2].

Cell‐penetrating peptides (CPPs) are cationic peptides with a maximum length of 30 amino acids that can induce the cellular uptake of nucleic acids, proteins and medicines that do not readily enter cells. CPPs have been extensively studied for their exceptional tissue penetration capability [70]. Significantly, a CPP‐drug combination has the ability to pass through the plasma membrane without requiring energy in many cell types [71]. Cationic polymers can bind to nucleic acids, evade the reticuloendothelial system (RES), resist enzymatic breakdown and successfully reach the nucleus [72]. CPPs, in contrast to other translocation delivery vehicles, have the ability to penetrate cells without causing damage to the cellular membranes, making them very effective and safe [73]. Nevertheless, the process by which CPPs enter cells is still not fully understood. The three documented processes are direct penetration, endocytosis and the creation of temporary membrane holes [74]. The specific mechanism by which CPPs exert their effects is contingent upon the characteristics of the cargo, cell type, membrane composition and peptide concentration [75].

Initially, CPPs were derived from naturally existing proteins that had already demonstrated exceptional translocation properties, such as HIV transactivator of transcription (TAT) and *Drosophila Antennapedia* transcription factor [76, 77]. Subsequently, a new series of CPPs with different sequences and forms have been developed, of which Pip [78, 79, 80, 81, 82], (RXRRBR)_2_XB (B peptide) [83, 84, 85] and (RXR)_4_XB [86, 87] are the most frequently used CPPs for drug delivery to muscle tissues. A peptide‐conjugated PMO (PPMO) designed to skip DMD exon 51 (SRP‐5051) has completed the dose‐finding phase of the MOMENTUM trial (NCT04004065), and a phase II study of SRP‐5051 is ongoing [88]. SRP‐5051 is based on eteplirsen conjugated to CPPs. Although eteplirsen is considered safe and shows potential functional advantages, its use remains controversial because of the limited synthesis of dystrophin. The limited effects of DMD drugs may be related to the limited amount of drug entering the cell. Researchers chemically linked an arginine‐rich B‐peptide from Sarepta Therapeutics to eteplirsen and administered the resulting PPMOs to canines. Strong dystrophin expression was observed in the skeletal muscles, specifically in the gastrocnemius and soleus muscles. Although SRP‐5051 enhances therapeutic efficacy in animals, it has exhibited toxicity such as hypomagnesaemia in human trials [89].

Dosage and treatment regimens are crucial for achieving maximum dystrophin expression while minimising the occurrence of side effects. Regrettably, whereas PPMO conjugation has optimised the delivery to the target [90], it has also facilitated penetration into organs, including the liver, which raises concerns over potential toxic side effects [91]. Administering PPMO at lower doses or with longer intervals between systemic injections is an easy and feasible method that could reduce side effects and potentially improve the cost‐effectiveness and patient‐friendliness of the treatment [91]. Moreover, CPPs could be used in combination with tissue‐targeting strategies for drug delivery to achieve both targeting specific tissues and enhanced penetration. The skeletal muscles of mdx mice treated with CPP‐muscle targeting ligand‐drug complex (B‐MSP‐PMO) revealed a restoration of dystrophin protein at a level of up to 25% of the normal level. In comparison, the B‐PMO conjugate exhibited a restoration of only approximately 10% of normal levels. It was shown that the B‐MSP‐PMO conjugate significantly improved dystrophin splice correction compared with B‐PMO and MSP‐PMO [92]. However, further investigation of the localisation and biological function of increased dystrophin needs to be done to confirm whether these elevated levels are biologically active before the approach could be tested in patients.

Notably, suitable aptamers are a versatile option as targeting ligands, as aptamers combine both the ability to target specific tissues and the ability to increase drug uptake in cells compared with peptide and antibody targeting strategies [93]. Cell‐Internalisation SELEX is a screening technology that uses cell‐based technology to find aptamers that can deliver cargo into the cytoplasm of target cells. In this novel selection strategy, surface‐bound aptamers are eliminated during the continuous selection process, whereas internalised aptamers are extracted for amplification [94], resulting in aptamers with both targeting and internalisation capabilities.

## 4 Discussion and Future Perspectives

Overall, each skeletal muscle‐targeting ligand has advantages and disadvantages (Table 1). As screening techniques for peptides and antibodies are well established, there are currently more studies focusing on skeletal muscle‐targeting peptides and antibodies. However, peptide and antibody ligands face additional hurdles owing to protease degradation. Although muscle‐targeting peptides have a decreased tendency for immunogenicity compared with antibody‐targeting techniques, their oral bioavailability remains low. Aptamers and small molecules exhibit high permeability, simple chemical production and low immunogenicity. However, small molecule ligands generally exhibit lower affinity because of their small size and high mobility. In contrast, aptamers can serve both targeting and internalisation functions and are novel and promising ligands for skeletal muscle‐targeting delivery systems.

**TABLE 1:** Ligands for specific targeting of skeletal muscle.

Type	Molecular weight	Immunogenicity	Stability	Modifiability	Example	Target	References
Antibody	Big	Intense	Poor, denatured under high temperature	Difficult, limited modification	mAb TfR1	TfR1	[95, 96, 97, 98]
					mAb 3E10	Myosin IIb	[32, 33]
Peptide	Medium	Moderate	Fair, stable at normal condition	Easy, wider variety of modification	ASSLNIA	Unknown	[40, 48, 49, 92, 99, 100, 101, 102, 103]
					M12	Unknown	[41]
					A2G80	α‐Dystroglycan	[42, 47, 104, 105, 106]
					FS2	Unknown	[43]
Small molecule	Small	Slight	Fair, stable at normal condition	Difficult, limited modification	C16‐HA	Unknown	[62]
					L‐carnitine	Na+‐coupled carnitine transporter (OCTN)	[63]
Aptamer	Medium	Slight	Good, stable under various conditions	Easy, wider variety of modification	A01B	Unknown	[64, 67, 68]
					HSM01	Unknown	[66]

From the perspective of ligand selection, when a drug requires a targeting ligand for the skeletal muscle, peptide ligands should be considered the primary choice. Peptides have the broadest applicability and have been studied extensively. When the drug to be targeted for delivery is AAV, almost all studies have used a targeting peptide for capsid modification of the AAV, and the transduction efficiency could increase by 10‐ to 50‐fold [52, 53]. Other viral vectors could be modified with protein and show an >8‐fold increase in transduction efficiency [58]. If the drug to be targeted for delivery is an oligonucleotide, peptides and antibodies may be good choices. From the available studies, targeting peptides and antibodies could achieve a similar exon‐skipping effect enhancements of about 10‐fold [41, 96]. In contrast, small molecules are associated with exon‐skipping enhancements of approximately 2–5 folds [62]. Nanoparticles are multifunctional and come in a variety of forms; thus, all four targeting strategies previously discussed are applicable for nanoparticle modification, yielding comparable targeted delivery outcomes [47, 98, 107].

In addition, if the action site of the cargo is located inside the cell and the cargo cannot pass through the cell membrane, the internalisation pathway becomes crucial. As mentioned above, the combination of targeted ligands and CPPs will be a major trend in the future, and the order of ligands, CPPs and drugs needs to be addressed. Yin et al. found that the CPP‐muscle‐targeting ligand‐drug complex (B‐MSP‐PMO) showed improved dystrophin splice correction compared with B‐PMO and MSP‐PMO. In contrast, when changing the order of CPP (B peptide) and muscle targeting ligand (MSP) to MSP‐B‐PMO, the MSP‐B‐PMO complex showed lower exon skipping activity and dystrophin restoration than B‐MSP‐PMO, and even lower than that of the B‐PMO complex [92]. Further investigations revealed that the increased cellular absorption of B‐MSP‐PMO into muscle cells resulted in higher levels of exon‐skipping activity compared with MSP‐B‐PMO. One possible explanation is that the cell‐penetration ability of the B peptide may be hindered by spatial resistance when the B peptide is located in the middle of the complex. Collectively, these data show that the orientation of ligands in a complex is a crucial factor in determining cellular absorption and activity when directly linked to the cargo.

The targeting ability of the ligand may be influenced by factors such as molecular weight and density. It is demonstrated that the average ligand binding affinities are not linear with molecular size by comparing the protein‐ligand binding affinities for over 8000 ligands with 28 protein targets [108]. Meanwhile, it has been found that dual‐targeting delivery systems may have better targeting efficiency than single‐targeting systems in the field of targeting tumours [109, 110]. Ligand densities also merit consideration in the design of targeting delivery systems, as increasing the density of targeting ligands may result in steric hindrance, which could impact the accessibility of the receptor and cause enlargement of the targeted ligands [111]. Although there is no available study focused on the number and combination of skeletal‐targeting ligands on the effects of targeting delivery system of cargos, these findings in cancer therapies emphasise the need of optimising ligand density and combination targeting in both in vitro and in vivo settings using imaging techniques and efficacy investigations.

Currently, most research on skeletal muscle‐targeting ligand development is based on the C2C12 or HSkMC cell lines. The immortalised cell lines such as C2C12 can be divided indefinitely, which makes them useful for long‐term studies. They also provide a more consistent and reproducible model. However, the genetic modifications used to immortalise these cells may alter their behaviour and characteristics, potentially affecting the validity of the results. In addition, they may not fully replicate the in vivo environment as accurately as primary cells. Primary skeletal muscle cells closely mimic the in vivo environment, making them highly relevant for the study of muscle physiology and disease. However, primary cells have a limited number of divisions and there may be significant variability between different batches. The use of induced pluripotent stem cells (iPSCs) might be explored in future studies. iPSCs can be generated directly from adult somatic cells, offering a promising avenue for personalised therapies [112]. They can differentiate into any cell type and can be expanded indefinitely. However, differentiation of iPSCs into skeletal muscle cells is complex and time‐consuming. With technological advances, cell models and new techniques that are closer to actual patient situations should be incorporated into the design of targeted ligand‐screening experiments.

Precise ligands that achieve selective targeting will also be the focus of future research. For example, heart failure is the leading cause of death in patients with DMD. For gene therapy drugs to have a meaningful impact on the course of the disease, it is crucial that they could be effectively delivered to both the skeletal and cardiac muscles. However, there are occasions when a distinction must be made between the skeletal and cardiac muscles. For example, systemic muscle relaxants have been shown to be statistically effective in the treatment of muscle spasms [113], and in order to increase the accumulation of relaxants in skeletal muscle and reduce the side effects on the heart, targeting ligands must then be designed to differentiate between skeletal muscle and cardiac muscle. Therefore, precise ligands should be considered in future studies.

Artificial intelligence (AI) can effectively assist in addressing and resolving these issues, thereby reducing the time and effort required. First, the traditional methods for identifying precise ligands are time‐consuming and costly. AI accelerates this process by analysing large datasets to identify promising candidates more efficiently [114]. AI, particularly machine learning (ML) and deep learning (DL), can predict and optimise the structure and function of targeting ligands. For example, ML and DL can be used to correlate the physicochemical properties of skeletal muscle‐targeting ligands, such as type, charge, molecular weight, polarity, and ligand‐to‐drug ratio, with disease parameters. This model can serve as a predictive tool to assist in the design of new skeletal muscle‐targeting drug delivery system formulations. Furthermore, AI models can predict the potential mechanisms of the screened targeting ligands, ensuring that the peptides bind effectively to skeletal muscle cells and enhance the precision of targeted therapies. AI is essential for advancing skeletal muscle‐targeted delivery, and there is an urgent need for future research in this area to enhance the precision, efficiency and personalised treatment strategies.

Early intervention is crucial in the management of skeletal muscle diseases. If skeletal muscle cells have already undergone extensive damage, fibrosis and transformation into adipose tissue, severe mobility impairment may have occurred and conventional interventions may have limited efficacy [115]. Molecular and genetic treatments, including gene therapy and ASO, focus on correcting genetic defects and are most effective when administered before significant muscle damage occurs. Advanced drug‐targeting delivery strategies offer promising solutions for mitigating the effects of late diagnosis. Targeting strategies could deliver therapeutic agents directly to the affected muscle tissues, ensuring higher drug concentrations at the site of action and higher therapeutic effects in a mouse model [41, 92]. This targeted approach not only enhances the efficacy of treatments but also increases the possibility of reversing muscle degeneration, even when intervention occurs later in the disease course.

In this review, we classify skeletal muscle‐targeting ligands into peptides, antibodies, small molecules and aptamers, describing their distinct characteristics, screening methods, research progress, and providing a detailed analysis of each strategy. In addition, we highlight the importance of internalisation in drug delivery systems. Essentially, there is no universal approach that is effective in all circumstances, and the selection of an appropriate ligand is entirely dependent on its specific purpose. Skeletal muscle‐specific drug delivery has great potential to avoid toxicity, improve efficiency and reduce costs; however, there is still relatively little research on muscle‐targeting ligands. Efforts should be made in the future to investigate more ligands that specifically recognise skeletal muscle, with the aim of enhancing the therapeutic effect and promoting patient well‐being.

## Author Contributions

Xiaofang Li collected the related papers and wrote the manuscript. Jintao Xu, Shanshan Yao and Ning Zhang revised the manuscript. Bao‐Ting Zhang and Zong‐Kang Zhang provided the ideas, funds and critical suggestions. All authors approved the final version of the manuscript.

## Conflicts of Interest

The authors declare no conflicts of interest.
